# Pharmacist-Led Prescribing in Austria: A Mixed-Methods Study on Clinical Readiness and Legal Frameworks

**DOI:** 10.3390/pharmacy13050130

**Published:** 2025-09-08

**Authors:** Olaf Rose, Clarissa Egel, Johanna Pachmayr, Stephanie Clemens

**Affiliations:** 1 Institute of Pharmacy, Pharmaceutical Biology and Clinical Pharmacy, Paracelsus Medical University, 5020 Salzburg, Austria; 2 Center of Public Health and Health Services Research, Paracelsus Medical University, 5020 Salzburg, Austria

**Keywords:** pharmacist, prescribing pharmacist, pharmacist-led prescribing, pharmacy practice, expanded role

## Abstract

In Austria, community pharmacists may dispense prescription-only drugs in exceptional emergency cases. Hospital pharmacists are permitted to adapt or discontinue therapy with prior physician approval. This mixed-methods study explores how Austrian pharmacists interpret and apply these frameworks, their readiness for expanded roles, and the systemic conditions required to support broader clinical engagement. A cross-sectional design was used with two online surveys targeting community and hospital pharmacists. Additionally, 15 semi-structured interviews were conducted (ten community, five hospital pharmacists). Quantitative data were analyzed descriptively; qualitative data were examined using Mayring’s content analysis. Data integration followed a triangulation design via mixed-methods matrices. A total of 238 community and 53 hospital pharmacists responded. Findings show that community pharmacists frequently apply clinical judgment in urgent situations and navigate regulatory grey zones. Over 88% support expanded roles, particularly in continuing contraceptives, managing chronic diseases, and treating infections using point-of-care testing. Hospital pharmacists report limited implementation of their framework, hindered by institutional inertia, staffing shortages, and poor access to patient data. Confidence in clinical pharmacotherapy decisions was limited. Targeted training and policy support are essential.

## 1. Introduction

In Austria, community pharmacies serve as a cornerstone of primary healthcare, ensuring the population’s access to medicinal products and providing a wide range of pharmaceutical services. Beyond the dispensing of prescription and non-prescription medicines, pharmacists are involved in numerous health-related tasks, including patient counselling, the preparation of magistral formulations, emergency medication supply, travel health advice, vaccination counselling, and contributions to public health initiatives such as screening programs [1]. This comprehensive scope reflects their critical role in facilitating safe and effective medicine use in outpatient care. By law, community pharmacists are responsible for ensuring that medication is dispensed in accordance with current pharmaceutical and medical standards. In most cases, this requires a valid medical prescription. However, since 1973, an exception has been in place that allows pharmacists to respond independently to patient needs in exceptional circumstances. Under a special clause of the Austrian Prescription Law (§ 4 Abs. 6, Rezeptpflichtgesetz), pharmacists are authorized, and obligated, to dispense prescription-only medication without a prescription in emergency situations [2]. This provision enables pharmacists to use their professional judgment in situations where immediate access to a physician is not possible, provided that the medicine is dispensed in the smallest available package and the situation meets specific criteria (e.g., on weekends, night shifts and general physicians’ off-peak hours). These typically include the urgency of treatment, the nature of the drug, and whether the patient is undergoing long-term therapy or is at risk of health deterioration without immediate access to drugs. Notably, pharmacists bear the responsibility for evaluating the emergency on a case-by-case basis and carry responsibility. Historically, this emergency dispensing authority has been reinforced through various legal instruments, which affirm the pharmacist’s duty to ensure continued supply of necessary drugs, particularly in urgent situations. Despite the vagueness of the legal term “emergency,” its interpretation is entrusted to pharmacists, offering them a degree of flexibility not commonly found in other areas of most healthcare systems. As such, this provision not only fills a regulatory gap but also positions pharmacists as autonomous decision-makers in critical, time-sensitive cases [3].

Austrian hospital pharmacists are involved in pharmaceutical manufacturing (e.g., cytostatic agents), logistics, procurement, and medication reconciliation at admission, and they often participate as members of the ward-based medical team, although this is not a mandatory responsibility. They collaborate closely with physicians and nurses, especially during key transitions of care such as hospital admission and discharge, where structured medication reviews are recommended in the Austrian structural plan for healthcare (ÖSG) as a quality criterion for inpatient care. Until recently, Austrian hospital pharmacists could make recommendations but not alter prescriptions independently. However, a major legislative development authorizes hospital pharmacists—within the framework of a medical order or protocol—to substitute drugs, adjust dosage forms and strengths, and discontinue or continue therapy autonomously. This “new provision” marks a significant step toward more collaborative and flexible models of pharmaceutical care, granting pharmacists increased legal security and the potential for more efficient workflows [4]. Its implementation, however, remains at the discretion of each hospital and the approvement of the medical head of the department, resulting in substantial variation in how, and whether, these new responsibilities are exercised.

In contrast to Austria’s cautious approach, international developments in pharmacist prescribing provide compelling evidence of broader professional roles. In countries such as the United Kingdom, Canada, and Australia, pharmacists have gained prescribing rights under structured frameworks that range from independent prescribing to collaborative or protocol-based models [5]. For example, pharmacists in the UK can qualify as independent prescribers authorized to prescribe any licensed medicine within their clinical competence. In Canada, prescribing authority varies by province but can include chronic disease management, therapeutic substitution, and treatment for minor ailments [6,7,8,9]. Recent regulatory changes in France reflect this trend as well: since June 2024, pharmacists have been authorized to dispense selected antibiotics without a prescription, provided that a pharmacy-based rapid diagnostic test yields a positive result and the pharmacist has completed a brief five-hour training program [10].

These international models suggest that pharmacist prescribing may enhance healthcare system efficiency, improve patient access to treatment, and reduce the burden on primary care services [11,12]. Studies have shown that pharmacist prescribers adhere closely to clinical guidelines, reduce medication errors, and achieve high levels of patient satisfaction, particularly in managing chronic conditions and minor ailments [13,14]. In regard of these international developments, Austrian pharmacists continue to operate within comparatively restrictive legal and structural boundaries. Given the recent legislative changes and ongoing global trends toward expanded pharmaceutical care roles, it is timely to assess how Austria’s existing provisions are applied in practice and whether there is readiness among pharmacists for broader clinical responsibilities. This study aimed to investigate the current use of expanded pharmaceutical responsibilities in Austria, including the application of the emergency dispensing clause in community pharmacies and the early implementation of expanded competencies in hospitals. It further explored pharmacists’ willingness and readiness to adopt extended roles, and the systemic prerequisites needed to support such developments via a mixed-methods approach.

## 2. Materials and Methods

This study employed a mixed-methods design integrating quantitative and qualitative data to comprehensively explore Austrian pharmacists’ perspectives on expanded clinical responsibilities. The methodology included two main components: digital surveys and in-depth semi-structured interviews, conducted with community and hospital pharmacists. The link for the survey for community pharmacists was distributed by the Austrian Pharmacists’ Association (Österreichischer Apothekerverband, Vienna, Austria), comprising pharmacy owners. In contrast to many other countries, pharmacy owners in Austria must be licensed pharmacists, may own only one pharmacy, and are closely involved in daily practice. The link for the questionnaire for hospital pharmacists was distributed by the Austrian Pharmacists’ Association (Vienna, Austria), Austrian Association of Hospital Pharmacists (AAHP, Vienna, Austria) and the Austrian Society of Hospital Pharmacy (KH Pharmazie, Vienna, Austria). This convergent approach enabled triangulation, allowing for a comparison and expansion of broad quantitative trends with in-depth qualitative insights, and was integrated in mixed-methods matrices [15]. In the mixed-methods matrices, selected interview quotes are used only to illustrate and contextualize survey findings; they are not intended to be representative of all participants.

### 2.1. Participants and Inclusion Criteria

Participants were registered pharmacists practicing in Austria, either in community or hospital settings. Eligibility was independent of professional experience, location, or employment status. All participants were required to be actively practicing at the time of data collection.

### 2.2. Digital Questionnaire Development and Validation

Two digital questionnaires were developed, one each for community and hospital pharmacists. For hospital pharmacists, two separate guides were created based on whether they had implemented the expanded competencies in their hospital or not. The questionnaires were structured around two domains: (1) clinical-practical application and (2) professional development. The development of the survey instruments followed best practices for instrument design as recommended in mixed-methods research frameworks by Creswell & Clark and followed a three-phase validation approach, including item generation, content and face validation, and legal review [16,17]. For methodological rigor, validation steps were aligned with standards for content validity as outlined by the COSMIN initiative [18]. Response scales were standardized across both surveys (e.g., Likert-like scales, beginning with the most positive option).

Except for one conditional “If-yes” question, all survey items were designed as mandatory resulting in consistently high response rates. Follow-up questions were displayed based on the previous selection. The questionnaires were administered in German language. They were distributed via the AAHP, and the KH Pharmazie. Data collection periods were 28 May to 23 June 2025 (community pharmacists), and 31 March to 13 June 2025 (hospital pharmacists). Participation was voluntary and anonymous. The questionnaires were digitalized in Google Forms (Google LLC, Mountain View, CA, USA) and the links were provided by email to the members of the associations.

#### Data Analysis

Survey responses were exported from Google Forms to Microsoft Excel (Office 365, Microsoft, Redmond, WA, USA). Descriptive statistics were calculated: means and standard deviations or medians and interquartile ranges for continuous variables, and frequencies and percentages for categorical variables. Visualizations such as bar charts were generated to illustrate key trends. To assess associations between variables, Spearman’s rank-order correlation was applied due to the ordinal nature of the data.

### 2.3. Interview Development and Validation

Interview guides were developed separately for community and hospital pharmacists, based on the same two domains used in the survey.

Content validation was conducted with experienced pharmacists out of both different settings. For community pharmacists, items were refined to assess familiarity with and application of the emergency clause, including frequency and strategies to mitigate patient risk. For hospital pharmacists, additional questions assessed the awareness and implementation of their expanded role, which needed to be cleared by a physician beforehand.

Face validation helped to establish clarity, neutrality, and the removal of redundant or irrelevant questions. It was performed with pharmacists and pharmacy assistants out of both settings to ensure clarity. Minor changes were made. Linguistic adjustments were made to facilitate clear and unbiased questioning.

Legal validation was conducted by a legal expert from the Austrian board of pharmacists (Vienna, Austria) and from a lawyer of the pharmacists’ association to ensure compliance with professional standards. Questions were modified accordingly.

Questions were posed in German language. Interviewees were recruited via expert referral and snowball sampling. We applied snowball sampling to identify participants with relevant expertise. While this approach may increase the likelihood of recruiting like-minded individuals, it is widely used in qualitative health services research and was chosen deliberately to ensure systematic access to informed participants while avoiding a pure convenience sample. Interviews were conducted in person or via phone, audio-recorded, and transcribed using the AI-based tool “aTrain” [Microsoft, Redmond, WA, USA version 1.3.0) [19].

#### Data Analysis

Transcripts were analyzed using MAXQDA (VERBI Software. MAXQDA 2022 [computer software], Berlin, Germany), employing Mayring’s method of qualitative content analysis [20]. Deductive categories derived from the interview guides were combined with inductively developed subcategories, enabling a structured yet flexible thematic analysis. Furthermore, a frequency count of categories was performed to identify the most commonly mentioned themes.

### 2.4. Ethics and Consent

Ethical approval was obtained from the Ethics Committee of the Paracelsus Medical University, Salzburg, Austria (PMU-EK-2024-0039), approval date: 4 February 2025. Written informed consent was secured from all interview participants. The study adhered to the Declaration of Helsinki, Good Clinical Practice (GCP), and General Data Protection Regulation of the European Union (Regulation (EU) 2016/679). Survey participation was anonymous, and consent was given electronically. All data were anonymized and stored securely on restricted-access servers.

## 3. Results

### 3.1. Qualitative Results

#### 3.1.1. Interviews with Community Pharmacists

Following snowball-recruiting, ten interviews with practicing community pharmacists in Austria were conducted, amounting to a total duration of approximately 2 h, 55 min, and 55 s.

Interviews with community pharmacists were mainly centered around the implementation of the emergency clause into clinical practice. Physician unavailability was the most frequently mentioned application context of the emergency clause. Pharmacists reported that the provision was particularly relevant when patients could not access a doctor in a timely manner. As one pharmacist noted:

*“The first big point is when no general practitioner is available or when waiting times are too long, and if I realize that this is exactly what the patient needs but it would take too long to get a doctor’s appointment.”*

(CP8, Pos. 13).

The emergency clause was also reported to be used during night shifts or emergency services, when medical services were limited. One pharmacist explained:

*“Especially during night shifts or on Sundays, you notice that it is needed more often.”*

(CP3, Pos. 8).

Another common scenario involved tourists or patients on holiday who forgot their drugs at home. For more than half of the interviewed pharmacists, the application of the emergency provision was perceived as clear, and they reported feeling legally secure in using it. One pharmacist explained:

*“I actually feel well protected because I’m always allowed to decide for myself what constitutes an emergency. And actually, I feel quite well protected in that regard.”*

(CP1, Pos. 13–14).

Some pharmacists did not consider discontinuation of chronic medication to be covered by the emergency clause, while others did. One frequently mentioned approach was checking the patient’s previous prescriptions based on health records. Another common strategy involved supplying the required medication and asking the patient to submit a valid prescription afterwards. As one pharmacist explained:

*“Often, the patient receives a temporary supply and later brings the prescription.”*

(CP9, Pos. 49).

Pharmacists emphasized their professional judgment when distinguishing between emergency and convenience. The patient assessment and symptoms were used as indicators. As one pharmacist stated:

*“It’s not that a patient just comes in and asks for something and immediately receives it. There’s always an assessment first.”*

(CP6, Pos. 49).

One pharmacist, for instance, stated: *“I never supply oral antibiotics without prescription because I think that really belongs in the hands of a physician.”* (CP8, Pos. 41). More than half of the pharmacists interviewed mentioned specific medication or therapeutic areas in which they could envision an expanded prescribing role. A recurrent theme across the interviews was the desire to independently manage the continuation of chronic medication, particularly for cardiovascular or metabolic diseases such as hypertension, diabetes, or hypercholesterolemia. One pharmacist noted:

*“…of course, for chronic medication. That would also be a huge relief for the entire healthcare system.”*

(CP9, Pos. 73).

Another frequently mentioned category was infectious diseases including urinary tract infections and eye infections for which pharmacists could imagine supplying antibiotics in clearly defined scenarios. For instance, one participant stated:

*“There are certain antibiotics where, for example, if someone has a bacterial infection, it would be good if we could treat them right away.”*

(CP2, Pos. 49).

Similarly, several pharmacists expressed frustration about the need to refer patients for eye infections when the outcome is often predictable:

*“It happens very often that someone has an eye infection, and we have to send them to the doctor, even though we already know they’ll come back with a prescription for Gentamicin eye drops. These are exactly the kind of situations where it would make sense.”*

(CP1, Pos. 65).

Pharmacists expressed frustration with the bureaucratic barriers when applying the emergency clause:

*“There should be simplified reimbursement procedures, especially when the supply is clearly in the patient’s interest.”*

(CP6, Pos. 115).

Several pharmacists perceived a lack of support from physicians and their associations. All interviewed pharmacists anticipated that allowing pharmacists to take on more responsibility would relieve the burden on physicians and emergency departments. As one participant stated:

*“That would certainly reduce the burden on doctors and emergency departments because many issues could be handled directly by us.”*

(CP1, Pos. 109).

Another pharmacist emphasized the broader societal benefit:

*“I definitely think it’s a win-win situation for everyone because doctors’ practices are less overwhelmed and where else do you get professional advice from a fully qualified academic without paying for it?”*

(CP5, Pos. 85).

Additionally, pharmacists highlighted that expanded roles could directly enhance patient safety. Several participants pointed out that gaps in chronic medication supply, incorrect prescriptions, or avoidable hospital visits could be minimized. One pharmacist noted:

*“It would definitely improve patient safety and reduce prescription errors because we often detect mistakes when patients are switched to new drugs, but old ones are still listed.”*

(CP4, Pos. 77).

Table 1 summarizes and interprets qualitative results from the in-depth interviews.

#### 3.1.2. Interviews with Hospital Pharmacists

In addition to the community pharmacists, semi-structured interviews were conducted with hospital pharmacists to explore the implementation of the new Austrian pharmacy act, which expanded the responsibilities of hospital pharmacists. Where this was not the case, reasons for non-implementation were explored, alongside a broader reflection on the current role and responsibilities of hospital pharmacists in Austria. In total, five interviews with hospital pharmacists were conducted. Interviews lasted in total 125 min and 32 s.

At the time of data collection (February to April 2025), none of the hospital pharmacists interviewed reported applying the new provision. While the regulation formally allows for dose adjustments or the discontinuation of medication with physician approval, implementation within hospitals had not progressed beyond traditional practices. As one interviewee explained:

*“We point out incorrect dosages to the prescribers so that they can adjust the orders, but everything still happens through medical consultation.”*

(HP3, Pos. 5).

The most frequently mentioned reason for non-implementation was the lack of internal procedures and coordination. All interviewees stated that no standardized process had yet been agreed upon. One pharmacist explained:

*“We actually haven’t discussed internally yet how we are going to approach this, and we also haven’t clarified it with the physicians.”*

(HP1, Pos. 9).

Another pharmacist added:

*“We’ve already developed a proposal for these procedures, but it hasn’t been approved by the physicians yet. It’s still under discussion.”*

(HP4, Pos. 15).

Answers imply that it might be difficult to promote the implementation of independent activities if physicians need to formally approve. Concerns about taking responsibility were frequently expressed, showing limited confidence in one’s own skills. One pharmacist stated:

*“I honestly don’t know how it would be regulated legally. Who would be responsible if a mistake was made by the pharmacist?”*

(HP5, Pos. 19).

Some pharmacists also highlighted educational limitations, noting that the current Austrian curriculum did not include training for prescribing roles:

*“Our education doesn’t really prepare us for prescribing responsibilities.”*

(HP3, Pos. 37).

The same pharmacist pointed out that the current cooperation with physicians already worked well and made autonomous action unnecessary:

*“Our cooperation with the prescribers works so well that we’re not dependent on carrying out changes independently.”*

(HP3, Pos).

Other pharmacists stated cautious optimism with acknowledgment of existing challenges, as one participant said:

*“Overall, I see it as a positive development, but I simply believe that in Austria it may still take a bit more time until it really works.”*

(HP5, Pos. 83).

However, the new provision was recognized as a big step forward:

*“Colleagues put in an incredible amount of effort and worked hard so that we could get this clause implemented. That’s also an enormously significant achievement for ward pharmacy in Austria.”*

(HP1, Pos.).

And:

*“Thank god … we managed to get it into the government’s program.”*

(HP2, Pos.).

Interviewed pharmacists were mainly positive on the regulatory change and believed it paves the path for the close future:

*“If we were to implement this, I don’t think there would be much resistance from the physicians.”*

(HP3, Pos.).

Others highlighted the importance of interprofessional collaboration and communication:

*“I would always coordinate with the physician.”*

(HP1, Pos. 53).

Another pharmacist added:

*“It’s a matter of mutual appreciation and recognizing each other’s competencies. Decisions should be made together as a multidisciplinary team.”*

(HP3, Pos. 77).

Regarding collaboration, one of the most frequently mentioned aspects was the relief of physicians and nurses in daily practice:

*“I believe the advantages that could arise would definitely be a relief in terms of workload for both nursing and medical staff.”*

(HP1, Pos. 61).

Several participants emphasized that prescribing authority could accelerate processes and help reduce avoidable errors. One pharmacist pointed out:

*“Sometimes we leave a note in the chart, but it gets lost. If we could just decide ourselves, it would make things easier for everyone involved.”*

(HP5, Pos. 67).

Another pharmacist added:

*“There are situations where pharmaceutical prescribing authority would help implement clinically relevant decisions faster.”*

(HP3, Pos. 85).

Participants also highlighted the capability of pharmacists to prevent drug-related problems such as incorrect dosing or harmful drug–drug interactions:

*“We are the medication experts, and we can definitely help prevent serious drug-related problems and high-risk situations.”*

(HP2, Pos. 43).

Several pharmacists also referred to the lack of formal specialization programs in Austria except in Vienna and called for more peer-to-peer learning and the establishment of professional circles:

*“We are currently trying to build specialist groups where we can educate each other because we don’t have specialized pharmacist training in Austria.”*

(HP2, Pos. 55).

Despite the ambitions, limited personnel resources were mentioned in multiple interviews. Pharmacists described being responsible for several wards or entire hospitals, thereby restricting their capacity to engage more deeply in clinical services. One interviewee noted:

*“If each of us only had to cover one ward, we would need five times as many colleagues as we currently have.”*

(HP3, Pos. 53).

Table 2 interprets and summarizes the findings; Table 3 identifies barriers and facilitators on implementation of the new regulatory provision.

### 3.2. Quantitative Results

#### 3.2.1. Questionnaire with Community Pharmacists

The questionnaire was completed by a total of 238 participants, corresponding to a response rate of 13.4%. The majority of respondents were female and with a mean long standing professional experience of 26.37 years (SD = 9.58), indicating a relatively consistent distribution of professional experience across the sample. Almost all participants (90.34%) indicated they were employed while 23 participants (9.66%) reported being self-employed (Table 4).

**Figure 1:**
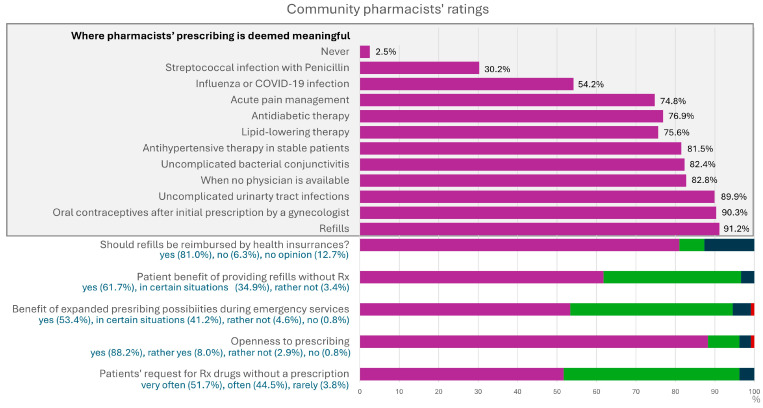
Results of the questionnaire for community pharmacists on expanded prescribing roles (*N* = 238).

Community pharmacists reported that they were frequently approached by patients requesting the dispensing of prescription-only medicines without a valid prescription. Most community pharmacists expressed a strong interest in an expanded role in prescribing, particularly when supported by point-of-care testing (POCT). The most endorsed scenarios for such a role included the renewal of prescriptions for chronic conditions such as hypertension, atherosclerotic cardiovascular disease (ASCVD), or diabetes, as well as the initiation of treatment for acute conditions such as urinary tract infections or conjunctivitis. Many pharmacists noted that the ability to prescribe would be particularly beneficial during emergency services, such as at night or on weekends. They specifically advocated for the authority to provide refills and for simplified reimbursement procedures. Detailed results of the questionnaire are presented in Figure 1.

Spearman’s correlation showed no significant association between age and lower openness to the expanded role in prescribing (Spearman’s Rho (ρ) = −0.115, *p* = 0.076, *N* = 238).

#### 3.2.2. Questionnaire with Hospital Pharmacists

The questionnaire was completed with a total of 53 participants. A response rate could not be provided, as the number of members of the two associations involved is unknown. Most respondents identified as female (*n* = 43, 81.1%). No respondents selected the non-binary option. With a mean age of 40.2 years and a professional experience of 13.7 years, they were younger and less experienced compared to their colleagues working in the community (mean age of 52.3 and 26.4 years of professional experience). Details on the demographics of the hospital pharmacists are displayed in Table 5.

**Figure 2:**
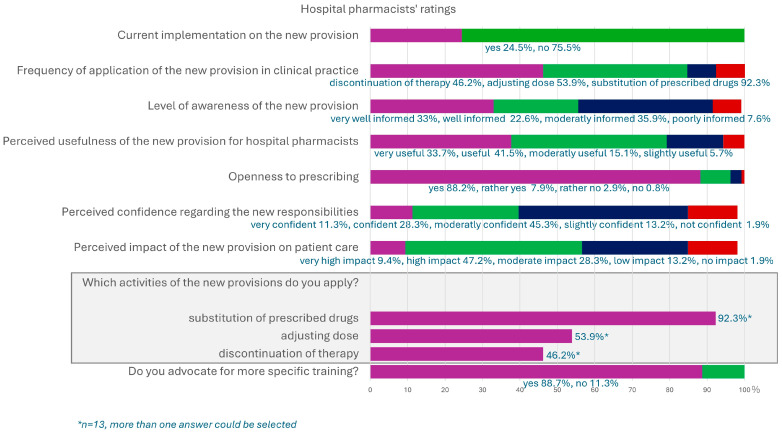
Results of the questionnaire for hospital pharmacists on expanded prescribing roles (*N* = 53).

All main survey questions were mandatory, resulting in full response rates for these items. If the question on current implementation of the new provision was answered with “yes”, a follow-up question on the specific activities was shown. This happened in *n* = 13 out of *N* = 53 participants. As a result, response rates for these items varied (Figure 2).

**Figure 3:**
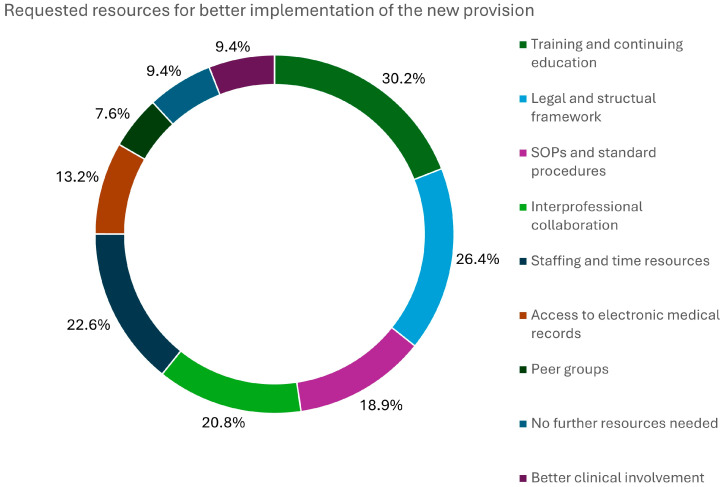
Requested resources for better implementation of the new provision on expanded prescribing activities in hospitals (*N* = 53).

Hospital pharmacists further specified what kind of additional resources they would prefer for a more successful implementation of the new regulations into their hospital setting. Better training and continuing medical education (CME) were chosen by almost every third clinical pharmacist (30.2%), indicating a need for more programs in this field. Clearance of the enhanced competencies by physicians is part of the request for a legal and structural framework (20.4%). The responses are displayed in Figure 3. Multiple resources could be selected.

In the Spearman’s correlation, there was a weak positive trend indicating that pharmacists with more professional experience felt somewhat more confident in handling the new responsibilities (Spearman’s Rho (ρ) = 0.219, *p* = 0.114, *N* = 53).

### 3.3. Mixed-Methods Approach

To explore pharmacist-led prescribing in Austria, data from qualitative interviews and the quantitative survey were integrated.

#### 3.3.1. Community Pharmacists

**Figure 4:**
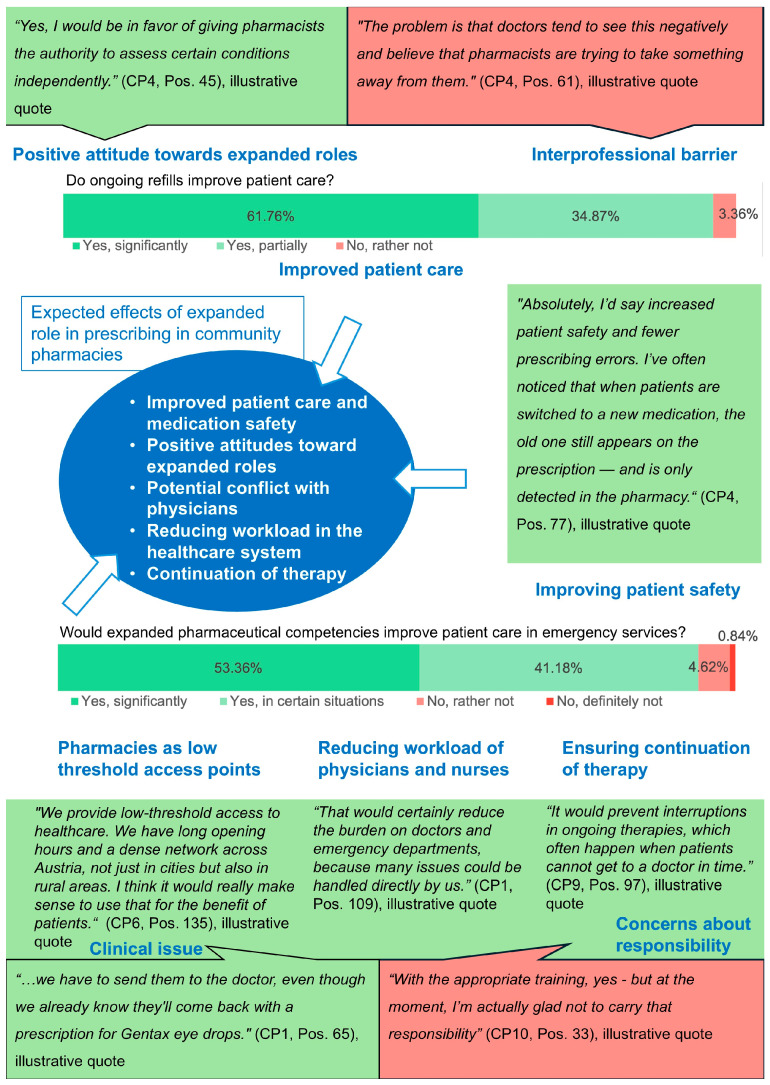
Mixed-methods matrix combining the results of qualitative and quantitative data on expanded roles in prescribing in community pharmacies. Qualitative statements are illustrative quotes drawn from interviews. They are not representative but serve to contextualize and underscore the quantitative survey findings.

While interviews focused on the practical application of the emergency clause, the survey emphasized pharmacists’ willingness to adopt expanded roles within the current regulatory framework. More than 90% of surveyed community pharmacists reported frequent patient requests for prescription-only medicines without a valid prescription, highlighting a demand beyond standard care. Consistently, eight out of ten interviewees indicated regular use of the emergency clause in daily practice. The survey also revealed strong support for pharmacist-led prescribing, particularly within clearly regulated and reimbursed models. In the interviews, nine out of ten pharmacists expressed confidence in managing defined indications and viewed an expanded role as particularly beneficial during emergency services at night and on weekends. Most pharmacists agreed that such reforms could ease the burden on the healthcare system and improve patient access. The mixed-methods matrix integrates findings from both data sources and draws conclusions (Figure 4).

#### 3.3.2. Hospital Pharmacists

**Figure 5:**
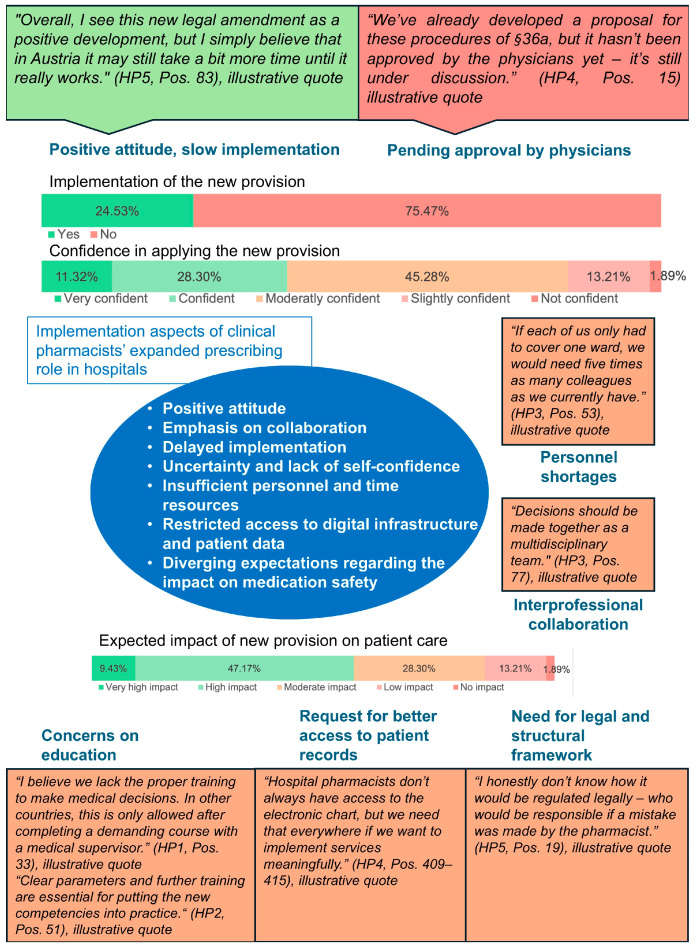
Mixed-methods matrix combining the results of qualitative and quantitative data on implementation of hospital pharmacists’ expanded roles in prescribing. Qualitative statements are illustrative quotes drawn from interviews. They are not representative but serve to contextualize and underscore the quantitative survey findings.

Over 75% of survey respondents stated that the expanded role had not yet been implemented in their hospitals, a finding echoed in all five interviews. A key barrier cited was the absence of standardized agreements with medical staff, where physicians have to formally assign the new competencies to the clinical pharmacists in their hospital, according to the new regulation. While half of pharmacists felt well or very well informed about the new provision, others have only limited awareness of their new competencies. Interviews confirmed this impression. About half of the pharmacists expected a clear impact of the new provision on patient care. At interviews, they mentioned faster response times and improved workflows. Others emphasized the importance of interdisciplinary decision-making. All interviewees agreed that the new provision represents a meaningful step toward clinical pharmacy advancement, though its success depends on time, experience, and structural adaptation. Limited personnel and time, lack of access to electronic records and patient data, and the importance of patient contact and restricted participation in ward rounds were emphasized as key barriers for successful implementation. The mixed-methods matrix shows the integration of results from qualitative and quantitative research (Figure 5).

This study explored the use of the emergency clause and the perspective of Austrian community pharmacists on an expanded role in prescribing, as well as the early implementation of a new provision on expanded prescribing by clinical pharmacists in hospital settings. By combining quantitative and qualitative data, the research offers nuanced insights into pharmacists’ professional practice and attitudes toward expanded prescribing roles.

## 4. Discussion

Community pharmacists in Austria apply the emergency clause with considerable diligence and confidence. The clause is designed for acute emergencies, grants pharmacists considerable flexibility in many situations, and is otherwise uncommon internationally, with a comparable provision existing in Poland, where a similar regulation has been established [21]. Pharmacists reported that they were aware of potential misuse and that they assessed every patient closely before using this option. Participating Austrian pharmacists, however, would prefer expanded prescribing rights like for instance in the UK and Canada, where pharmacists are entrusted with bridging prescription gaps to ensure medication adherence [22]. Survey results demonstrated that more than 95% of responding Austrian community pharmacists are open to expanded prescribing responsibilities. The qualitative interviews added depth to these findings, showing that the currently applied emergency clause is not only regarded as a legal safeguard, but also framed by pharmacists themselves as part of their professional responsibility towards patients. Taken together, these results point to a profession that is both willing and prepared to take on an expanded role in medication management. Importantly, this perspective is not unique to Austria but resonates with broader international trends. In the Netherlands, for example, over 85% of pharmacists indicated support for limited independent prescribing models, suggesting a comparable openness to redefining professional boundaries [23]. Similar attitudes were reported in Italy, where both pharmacists and patients expressed strong approval of pharmacist prescribing when clearly defined frameworks and safeguards were in place [24]. Our findings suggest that Austrian pharmacists, too, are prepared to assume more responsibility, particularly in stable, well-defined scenarios such as contraceptive refills or chronic disease maintenance. Thus, the Austrian case, while grounded in a specific legal context through the emergency clause, mirrors wider European developments and highlights a shared trajectory towards greater pharmacist involvement in prescribing. Nevertheless, the willingness to prescribe varied by indication. While oral contraceptives received near-universal support (90.3%), bacterial infections such as streptococcal tonsillitis were seen as more complex and required additional safeguards. This differentiation mirrors results from Australia and the UK, where pharmacists favored defined conditions and protocols, particularly when antibiotics were involved [25]. The legal obligation for continuing education, introduced in Austria in 2023 (in effect since July 2024), represents a foundational step for further role expansion [26]. Yet, participants stressed that formal training alone is insufficient. Adequate structures, clear reimbursement pathways, and legal protection were considered necessary to embed these expanded roles into daily practice. The findings should be interpreted with caution, as the response rate was 13.4% and thus the majority of pharmacy owners did not participate. Nevertheless, response rates of this magnitude are common in surveys among health professionals, and non-response bias cannot be entirely ruled out.

In contrast to the community setting, a new provision on expanded prescribing roles of clinical pharmacists, introduced in 2024, has seen limited uptake in hospitals. Over 75% of participating hospital pharmacists reported that the provision was not yet implemented in their institutions. Qualitative interviews confirmed the absence of clearance by physicians, standard operating procedures, interprofessional agreements, and practical training as primary barriers. The data illustrate that although legislation has created new opportunities, implementation lags significantly behind. This pattern reflects findings from other countries where similar reforms were introduced. For example, in the UK’s national health service (NHS) system, the introduction of pharmacist prescribers required years of coordinated institutional alignment and educational infrastructure before becoming routine [27]. Confidence levels among participating Austrian hospital pharmacists remained low. Nearly 60% reported moderate to no confidence in using the new provision, citing a lack of clinical integration and limited access to patient data. This mirrors the international experience, where pharmacist-led interventions were successful only when embedded in interdisciplinary teams and supported by interoperable digital systems [28]. Despite this, hospital pharmacists viewed the reform as a positive step. Many highlighted the potential of the new provision to strengthen their clinical role in dose optimization and therapy monitoring. However, they emphasized that pharmacist-led decisions must remain embedded in collaborative care models. This reflects a consensus in the literature that clinical pharmacists should definitely prescribe but complement—not replace—physician-led care in hospital settings, reflecting interprofessional collaboration as a key to better care [29,30,31].

A recurring theme was the need for peer exchange, structured case discussions, and institution-wide protocols to foster confidence and ensure consistency. Some participants called for a centralized support framework, perhaps coordinated by the Austrian associations of hospital pharmacy, to facilitate implementation and share best practices across hospitals.

The comparison between community and hospital settings reveals substantial differences in the maturity and integration of expanded pharmacist roles. The emergency clause has become an established and appreciated part of community pharmacists’ professional toolkit. By contrast, the new provision on the expanded role of the hospital pharmacist has not yet transitioned to operational practice. Community pharmacists work independently and directly interface with patients, which facilitates the autonomous use of legal tools. Hospital pharmacists operate in more hierarchical and interdisciplinary environments, where any expansion of responsibilities requires consensus, infrastructure, and clear protocols.

Yet, the findings also reveal important similarities. Across both settings, pharmacists emphasized the need for legal clarity, targeted education, and institutional support. Regardless of practice setting, the safe implementation of expanded roles depends on integrated training and shared responsibility models. These requirements reflect those identified in countries such as Canada, where pharmacist prescribing was successfully adopted only after comprehensive policy and educational reforms.

Taken together, the study highlights that Austria is at a pivotal moment in redefining the pharmacist’s role in healthcare. While community pharmacists are already applying expanded responsibilities under the emergency clause, hospital pharmacists are cautiously preparing for a shift that will require legal and systemic alignment.

## 5. Limitations

This study has several limitations. The qualitative subsample, particularly the five hospital pharmacists, was small and therefore cannot capture the full diversity of Austrian hospital settings. These findings should be regarded as exploratory rather than evidence of theoretical saturation. However, when interpreted alongside the survey results, the interviews provide valuable context and help to illustrate potential barriers and perspectives in greater depth; the aim was analytical insight rather than representativeness. Future studies should increase sample size and diversity, including underrepresented groups in this study, such as employed pharmacists and those not affiliated with professional bodies. Recruitment through the Austrian Pharmacists’ Association and hospital pharmacy networks may have introduced selection bias, potentially favoring more engaged or reform-minded participants. Additionally, the reliance on self-reported data could be affected by social desirability, especially concerning legal compliance or professional confidence. The dynamic nature of the new provision also presents a limitation. Since the provision was recently introduced, its implementation has been evolving. Institutional uptake, policy refinements, and professional consensus are still developing and could substantially change the observed patterns. Longitudinal studies will be required to assess the provision’s practical relevance over time. Our study did not explore pharmacists’ perspectives on the new requirement for mandatory continuing education introduced in 2023. As data collection occurred shortly after its implementation, pharmacists had limited experience with the regulation; however, this remains an important direction for future research to assess its adequacy in preparing for expanded prescribing roles. Despite these limitations, this study contributes original and practice-oriented insights into pharmacists’ readiness and the conditions required for meaningful role expansion in both ambulatory and hospital care.

## 6. Conclusions

This study highlights the evolving role of Austrian pharmacists in light of recent legal developments concerning expanded prescribing responsibilities in both community and hospital settings. While community pharmacists have long applied the emergency clause to ensure continuity of care and address acute access issues, hospital pharmacists are only beginning to explore the responsibilities introduced by the new provision. The findings reveal strong professional openness in both settings, but also point to critical structural, legal, and educational prerequisites that must be addressed to ensure safe and confident implementation. Community pharmacies and patients would benefit if the legislator were to amend the prescribing regulation to clearly define the scope of pharmacist-led prescribing in chronic diseases and acute infections. Guidelines and standards should subsequently be developed by the board of pharmacy to ensure consistent and safe application in practice. In contrast, participating hospital pharmacists require greater institutional support to operationalize their new role, including local approval procedures and formal integration into clinical teams. Their professional confidence should be strengthened through targeted pharmacotherapy training and clearly defined responsibilities within interprofessional settings. As pharmacy practice continues to evolve towards more patient-centered and autonomous models, these findings underscore the need for a supportive legal and institutional framework that enables pharmacists to fully contribute to care delivery. Future research should investigate the long-term implementation and clinical outcomes of expanded pharmacist roles to inform policy and strengthen the evidence base for clinical pharmacy in Austria and beyond.

## Figures and Tables

**Table 1:** Community pharmacists’ perceptions on using the emergency clause in dispensing prescription drugs, which allows them to dispense prescription drugs in certain situations and an expanded role (*N* = 10).

Item	Interpreted and Clustered Content of Interviews
Frequency of using the emergency clause (*N* = 10)	Several times per week (*n* = 8), rarely (*n* = 2)
Reported contexts for applying the emergency clause (*N* = 10)	Patient on holiday, forgot his drugs (*n* = 3), emergency services (*n* = 6), absence of a prescriber/physician (*n* = 9), acute medical emergency (*n* = 4)
Perceived legal confidence of the emergency clause (*N* = 10)	Very clear (*n* = 6), grey area/unclear (*n* = 4)
Examples of drugs frequently dispensed under the emergency clause (*N* = 10)	Chronic medication: antihypertensives, cholesterol-lowering drugs, asthma spray, anticoagulants, oral contraceptives Acute medication: antibiotics (oral, eye drops/ointments), pain medication, antipyretics, asthma spray
Decision-making strategies in applying the emergency clause (*N* = 10)	Reference to prior prescription (e.g., electronic medication record) (*n* = 5), provision with follow-up prescription requested (*n* = 4), credibility and coherence of patient’s information (*n* = 8), pharmaceutical judgment based on clinical assessment (*n* = 4)
Perceived limits of the emergency clause by pharmacists (*n* = 7)	Oral antibiotics (*n* = 1), use in small children and infants (*n* = 2), drugs with high potential for misuse (*n* = 5), psychotropic drugs and narcotics (*n* = 3)
Readiness to take on expanded responsibilities (*N* = 10)	Openness to expanded professional role (*n* = 9), lacking specific knowledge, being concerned about responsibility (*n* = 1)
Areas for expanded prescribing roles identified by pharmacists (*N* = 10)	Chronic medication (*N* = 10), oral contraceptives (*n* = 3), infectious diseases (*n* = 7), supplements (vitamin D, magnesium, *n* = 3)
Diagnostic options mentioned by pharmacists (*n* = 9)	Urine tests to confirm urinary tract infections, inflammation markers (e.g., C-reactive protein), rapid tests for infections (*n* = 9)
Suggestions for structural improvements related to the emergency clause (*n* = 2)	Dispensing a clinically appropriate package size (instead of the smallest available) (*n* = 2), uncomplicated reimbursement with health insurance (*n* = 2)
Perceived barriers to expanding pharmacists’ competencies (*N* = 10)	Resistance from physicians’ associations (*n* = 7), lack of resources (*n* = 4), medical educational concerns (*n* = 2)
Anticipated positive effects from expanding pharmacists’ competencies (*N* = 10)	Participants anticipated a reduction in workload for physicians and emergency departments (*N* = 10), enhancements in patient safety and continuity of care (*n* = 9), and improvements in public perception and professional satisfaction (*n* = 3)

**Table 2:** Summary of the content analysis of interviews with hospital pharmacists in Austria regarding the implementation of the amended provision permitting independent action on prescription medicines (*N* = 5).

Item	Interpreted and Clustered Content of Interviews
Implementation of the new provision in hospitals (*N* = 5)	No implementation so far but under consideration in one hospital. Currently, all recommendations need to be approved by physicians.
Reported reasons for deferred implementation (*N* = 5)	Uncertainty about procedure, concerns on being held responsible, lack of trust in own ability, lack of personnel resources.
Pharmacists’ desired level of autonomy (*N* = 5)	Expanded role and expectations perceived heterogeneous among participants.
Reflections on the implementation of the new legal authority (*N* = 5)	Interdisciplinary decision-making remains essential, collaboration more important than prescribing rights.
Appraisal of the new authority (*N* = 5)	Positive but cautious attitude, perceived as a big step towards professional development and implementation of clinical pharmacists. Recognition of efforts of clinical pharmacists’ associations.
The new provision seen from the interprofessional perspective (*N* = 4)	Acceptance by physicians questionable, communication and mutual respect are regarded as essential prerequisites for successful implementation.
Effects of expanded competencies (*N* = 5)	Relief on workload of physicians and nurses, faster clinical decision-making, improved patient safety and quality of therapy.
Recommendations for successful implementation of expanded clinical prescribing roles (*N* = 5)	Necessity of specialized training, standardizing processes, learning by doing, getting involved with the new competencies.

**Table 3:** Identified barriers on implementing the expanded role of hospital pharmacists (*N* = 5).

Aspect	Barrier	Facilitator
Clinical insights (*N* = 5)	Lack of direct patient contact (e.g., no ward rounds, reliance on files only)	Direct involvement in clinical decision-making (e.g., participation in ward rounds, follow-up meetings)
Infrastructure and access (*n* = 2)	Inconsistent access to electronic records	Full access to digital systems across all wards and institutions
Personnel resources (*n* = 2)	Understaffing	Increased staffing and more time for clinical services and patient contact
Clinical education and specialization (*n* = 2)	Reluctance to take on responsibility	Strengthened clinical orientation beginning at university level
Interprofessional collaboration (*n* = 2)	Skepticism among medical staff toward the pharmacist’s expanded role	Joint decision-making and shared responsibility models with physicians and nurses

**Table 4:** Demographic data of community pharmacists, who participated in the questionnaire (*N* = 238).

Parameter	Demographic Data
Gender	Female: *n* = 131 (55.0%)
	Male: *n* = 103 (43.3%)
	Non-binary: *n* = 2 (0.8%)
	Prefer not to say: *n* = 2 (0.8%)
Age	$\bar{x}$ 52.3 years (SD 9.6 years), median 54.0 years
Years of professional experience	$\bar{x}$ 26.4 years (SD 9.6 years), median 27.5 years
Employment status	Self-employed: 215 participants (90.3%)
	Employed: 23 participants (9.7%)

**Table 5:** Demographic data of hospital pharmacists, who participated in the questionnaire (*N* = 53).

Parameter	Demographic Data
Gender	Female: *n* = 43 (81.1%)
	Male: *n* = 10 (18.9%)
Age	$\bar{x}$ 40.2 years (SD 9.5 years), median 37.0 years
Years of professional experience	$\bar{x}$ 13.7 years (SD 9.4 years), median 12.0 years

## Data Availability

The individual questionnaires and all data are available from the corresponding author upon reasonable request.

## Abbreviations

The following abbreviations are used in this manuscript:

AAHP	Arbeitsgemeinschaft österreichischer Krankenhausapotheker
ASCVD	Atherosclerotic Cardiovascular Disease
CME	Continuing Medical Education
COSMIN	COnsensus-based Standards for the selection of health Measurement INstruments
EK	Ethics Committee
EU	European Union
GCP	Good Clinical Practice
NHS	National Health Service (UK)
POCT	Point-of-Care Testing
SD	Standard Deviation
UK	United Kingdom
KH Pharmazie	Österreichische Gesellschaft für Krankenhauspharmazie
